# Temporal course of attention bias toward emotional faces in individuals with autistic traits: an eye-movement study

**DOI:** 10.3389/fnins.2023.1218595

**Published:** 2023-07-28

**Authors:** Chunyan Meng, Taolin Li, Jing Wang

**Affiliations:** ^1^Department of Public Curriculum, Zigong Vocational and Technical College, Sichuan, China; ^2^Key Laboratory of Applied Psychology, Chongqing Normal University, Chongqing, China; ^3^Mental Health Guidance Center, Qianjiang Senior High School, Chongqing, China; ^4^School of Health Rehabilitation, Zigong Vocational and Technical College, Sichuan, China

**Keywords:** autism spectrum disorder, autistic traits, attention bias, temporal course, eye movement

## Abstract

**Introduction:**

Similar attention patterns have been found in individuals with autism spectrum disorder (ASD) and autistic traits (ATs). The Intense World Theory and previous studies suggest that individuals with ASD may demonstrate a vigilance-avoidance attention pattern toward emotional faces. However, the attention patterns in individuals with ATs remain unclear. Therefore, this study employs eye-tracking technology to examine the characteristics and temporal course of attention bias toward emotional faces in individuals with ATs.

**Methods:**

The Autism-spectrum Quotient (AQ) was used to evaluate the level of ATs among 2,502 college students. A total of 50 participants were selected from the 2,502 college students: 25 high-AQ group participants were randomly selected from the 10% of individuals with the highest AQ scores. Similarly, 25 low-AQ group participants were randomly selected from the 10% of participants with the lowest AQ scores. All selected participants completed an eye-tracking study while performing a dot-probe task with emotional faces (positive-neutral, negative-neutral, and negative–positive). By analyzing data from different time periods, the attention bias and time course of individuals with ATs toward emotional faces were investigated.

**Results:**

The results show that compared to the low-AQ group, the high-AQ group detected negative faces faster in the early stages of emotional face processing. As the presentation time of emotional faces increased (at the 2–3 s mark), the fixation scores for negative-neutral faces of the high-AQ group were less than 0.5, which was significantly lower than those of the low-AQ group. Meanwhile, the high-AQ group showed brief attentional avoidance toward positive emotion at 3–4 s in the positive-neutral trials, indicating that the high-AQ group exhibited attention avoidance to both negative and positive faces during the middle and later stages of emotional processing.

**Conclusion:**

This study suggests that individuals with ATs display a vigilance-avoidance pattern toward emotional faces. It contributes to a deeper understanding of the mechanisms of attention in persons with ATs and further supports the Intense World Theory.

## 1. Introduction

Autism spectrum disorder (ASD) is a pervasive neurodevelopmental disorder characterized by abnormal patterns of social interaction and communication, restricted interests, and repetitive and stereotyped behaviors (American Psychiatric Association, 2013). The Diagnostic and Statistical Manual of Mental Disorders, Fifth Edition (DSM-5) has added sensory hypersensitivity or insensitivity to the criteria for restricted interests and repetitive behaviors, considering perceptual abnormalities as one of the typical features of individuals with ASD (American Psychiatric Association, 2013). Autistic traits (ATs), which are a set of primary symptoms associated with ASD, are continuously distributed in the general population (Groot and Strien, 2017). The continuous distribution of ATs suggests that there are quantitative differences in the degree to which individuals possess ATs; nevertheless, individuals with ASD have higher levels of ATs compared with individuals with ATs in the general population (Lai et al., 2013; Murray et al., 2014). The Autism-spectrum Quotient (AQ; Baron-Cohen et al., 2001) is widely used to measure the level of ATs, and individuals with high AQ scores who do not meet the clinical diagnostic criteria for ASD can be referred to as individuals with ATs (Poljac et al., 2013; Li et al., 2020). Research has found that individuals with ATs and those with ASD share similar genetic and biological characteristics (Robinson et al., 2016; Bralten et al., 2018). Both groups exhibit impairment in social interactions, empathic abilities, and sensory processing (Robertson and Simmons, 2013; Takahashi et al., 2013; Guan and Zhao, 2015).

Individuals with ATs exhibit some attentional characteristics toward faces that are similar to those exhibited by individuals with ASD. Meta-analytic studies have shown that individuals with ASD gaze less toward the eyes compared with individuals without ASD and ATs, demonstrating an attentional avoidance toward the eye region (Papagiannopoulou et al., 2014; Hao et al., 2018). Studies on individuals with ATs have found that they gaze less at others’ eyes and display less visual search during face-to-face and video interactions (Freeth et al., 2013; Vabalas and Freeth, 2016). Meanwhile, individuals with ATs show less eye contact with others while watching videos (Chen and Yoon, 2010). In addition, previous studies have found that individuals with ASD do not exhibit attention bias toward positive (happy) emotional faces (Monk et al., 2010; Hollocks et al., 2016; García-Blanco et al., 2017). Similarly, individuals with ATs show fewer gaze orientation effects toward happy faces (Lassalle and Itier, 2015), and their reaction times to angry faces are faster (Folz et al., 2023). However, some researchers have also indicated that ATs in the general population do not affect the visual processing of emotional faces (Greene et al., 2020) or attentional bias toward fearful faces (Miu et al., 2012).

The Intense World Theory (Markram and Markram, 2010) posits that individuals with ASD exhibit excessive perception, attention, and emotional responses to sensory information. Excessive sensory information leads individuals with ASD to be in a state of information overload and to experience excessive fear and anxiety, thereby avoiding normal social and emotional communication (Sahuquillo-Leal et al., 2023). Therefore, according to the Intense World Theory (Markram and Markram, 2010), individuals with ASD may exhibit a vigilance-avoidance attention pattern toward emotional faces, in which they detect emotional faces more quickly in the early stages of processing due to overly intense perception and attention but may avoid emotional faces in the later stages due to sensory and emotional information overload. Some results suggest that individuals with ASD exhibit attention biases toward emotional faces (Monk et al., 2010; Chen et al., 2011; Hollocks et al., 2016), show a preferential response to negative emotional faces, and can detect angry faces faster than individuals without ASD or ATs (Chen et al., 2011; Isomura et al., 2014). More importantly, as the stimulus presentation time increases, individuals with ASD may exhibit avoidance of negative or threatening emotional faces (anger; García-Blanco et al., 2017; Ghosn et al., 2019). A segmented analysis was conducted on the attention time of individuals with ASD toward emotional faces, and it was found that the attention time toward negative emotional faces was significantly shorter than that of the control group during the last 3–4 s of the emotional face presentation (White et al., 2015), indicating a decrease in attention toward negative emotional faces in the later stages. These studies indirectly support the view of the Intense World Theory, suggesting that individuals with ASD may exhibit a vigilance-avoidance attention pattern toward emotional faces.

In summary, previous studies have shown that individuals with ASD exhibit vigilance–avoidance attention patterns toward emotional faces, but the attention pattern in individuals with ATs remains unclear. Our study uses a dot-probe paradigm combined with eye-tracking technology; presents positive-neutral, negative-neutral, and negative–positive emotional face combinations for 8 s; and analyzes eye-movement data at different time intervals. Through this paradigm and analytical method, we aim to address the following research questions. First, this study investigates the time course of attention bias toward emotional faces in individuals with ATs, which has rarely been explored in previous investigations. Second, we examine whether the overload of sensory and emotional information in these individuals could result in significant avoidance behavior. According to the Intense World Theory (Markram and Markram, 2010), our study proposes the following hypotheses. First, individuals with ATs exhibit attentional vigilance to emotional faces and detect negative expressions earlier than positive expressions. Second, as the presentation time of emotional faces increases, individuals with ATs may exhibit attention avoidance earlier due to emotional information overload.

## 2. Materials and methods

### 2.1. Participants

A total of 2,502 college students were recruited to complete the Mandarin version (Liu, 2008) of the AQ questionnaire (Baron-Cohen et al., 2001). Their scores were used to estimate their ATs. Then, 25 participants were randomly selected from the 10% of students with the highest AQ scores and identified as the high-AQ group. A further 25 participants were randomly selected from the 10% of students with the lowest AQ scores and identified as the low-AQ group. Table 1 shows the age variables and AQ scores for both groups. All the participants were required to be right-handed, to have normal or corrected-to-normal vision without color blindness or color weakness, and to be without a psychiatric disorder diagnosis.

**Table 1:** Ages and AQ scores of high-AQ and low-AQ group participants.

Group	Age (year)			AQ score		
	*M ± SD*	*t*	*p*	*M ± SD*	*t*	*p*
High-AQ	20.40 ± 1.56	0.806	0.424	28.08 ± 1.65	28.529	﹤0.001
Low-AQ	20.88 ± 2.53			13.44 ± 1.96		

AQ, Autism Spectrum Quotient. *p*-values and *t-*values were obtained from independent samples *t*-tests performed on ages and AQ scores between high-and low-AQ groups participants.

In accordance with the Declaration of Helsinki, all participants provided free and informed written consent before the experiment, and all procedures were approved by the research ethics committee of Chongqing Normal University. The procedures were performed in accordance with the current ethical guidelines and regulations issued by this committee.

### 2.2. Materials

A total of 18 pictures of the faces of six models with three different expressions (positive/happy, neutral, negative/sad) were selected from the Chinese facial affective picture system (Bai et al., 2005). There are three combinations of expressions, namely, positive-neutral pairs, negative-neutral pairs, and negative–positive pairs, and each combination was created from different expressions of the same person. A total of 40 college students (20 men) who did not participate in the experiment were recruited to score the valence (1 = extremely unhappy, 9 = extremely happy) and arousal (1 = very peaceful, 9 = very excited) of the facial pictures using 9-point Likert scales. There were significant differences in valence between the three types of expressions [*F*(1, 38) = 93.85, *p* < 0.001, η^2^_p_ = 0.71]; the valence of the positive pictures (7.07 ± 0.57) was significantly greater than that of the neutral (4.51 ± 0.48) and negative (2.89 ± 0.53) pictures. No significant differences were found for arousal [*F*(1, 38) = 3.51, *p* = 0.056, η^2^_p_ = 0.08; positive: 5.67 ± 0.43, neutral: 4.88 ± 0.16, negative: 5.69 ± 0.93].

### 2.3. Design

The experiment adopted the dot-probe paradigm. It was a two-factor mixed experimental design of 2 (Group: high-AQ, low-AQ) × 3 (Expression: positive-neutral, negative-neutral, negative–positive). Group, as a between-subjects factor; expression, as a within-subjects factor; response time; accuracy; and eye-movement data were dependent variables. The gender of the model in the picture and the location of the detection point were control variables.

### 2.4. Equipment

Recordings of eye movements were obtained using a Tobii Pro X3-120 eye tracker. E-Prime 3.0 was used to compile the experimental program and record the behavioral data. The Tobii Pro X3-120 eye tracker samples gaze coordinates at 120 Hz, and the monitor was set at 1,920 × 1,280 resolution. Tobii Pro X3-120 eye tracker was connected and communicated with E-Prime 3.0 via E-Prime Extension for Tobii Extension Packs; the experiments were conducted using E-Prime to drive the eye-movement device and complete the experimental stimuli and eye-movement data collection. When the experimental stimuli were presented, E-Prime managed the calibration of the participants and sent instructions to the recording software of the Tobii eye tracker to control the start and end of the recording while transmitting relevant experimental design information to the recording software of the Tobii eye tracker.

### 2.5. Procedures

Participants arrived at a quiet and soundproof laboratory, where the temperature was suitable; they were then seated approximately 70 cm from a 28-inch monitor, which was equipped with the Tobii Pro X3-120 eye tracker. Face picture size was 260 × 300 pixels (width × height), and the distance between the inner edges of the two faces was 11 cm, corresponding to a visual angle of 15.7° × 14.1°. After a short 9-point calibration of the eye tracker, participants completed the experiment.

**Figure 1:**
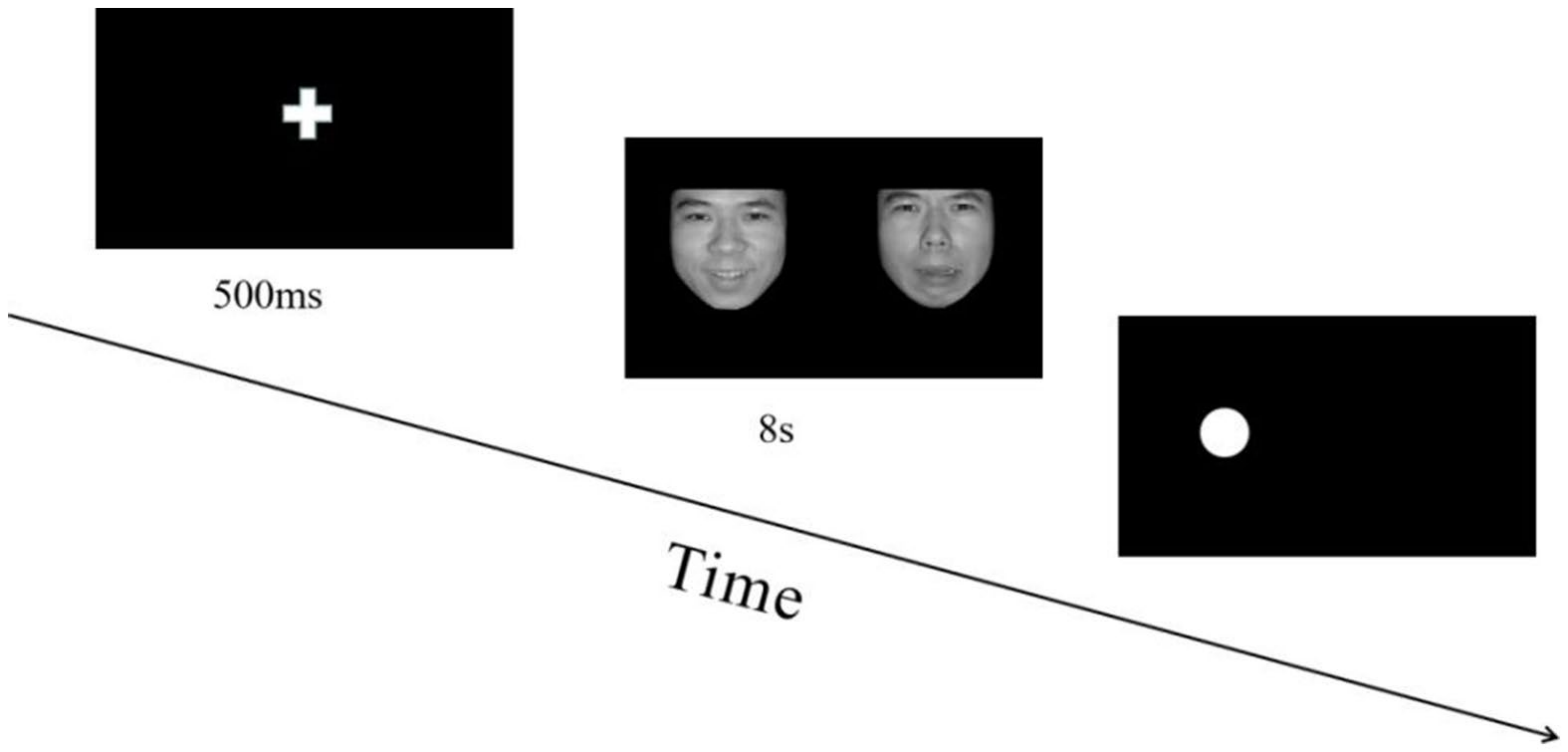
Flowchart describing the experimental design. All pictures in the experiment were selected from the Chinese facial affective picture system (Bai et al., 2005). During each trial, the order in which emotion combinations (positive-neutral, negative-neutral, negative–positive) were presented was random, and the positions of different emotion pictures in each combination were balanced.

The experiment included 72 trials and consisted of two blocks. In each block, three expression combinations were presented randomly (positive-neutral, negative-neutral, negative–positive). Participants could take a break between the two blocks and press the space key to start the next block. Prior to the experiment, each participant conducted a training session of approximately six trials, which could be repeated until the participant was familiar with the experimental procedure, and the accuracy was more than 80%. During the presentation, participants saw a centered white fixation cross on a black background for 500 ms and then emotional faces for 8 s simultaneously on the left and right sides of the screen. A probe point was then randomly presented on the left or right side of the screen, and the “1” key was pressed if the probe point appeared on the left side and the “2” key was pressed if it appeared on the right side. Participants were instructed to respond as quickly as possible while avoiding errors. After 200 ms of blank screen, the next trial commenced. An experiment trial is displayed in Figure 1.

### 2.6. Measurements and analyses

#### 2.6.1. Behavioural measurements

In the dot-probe task, this study mainly analyzed participants’ response time (RT) in the congruent (probe points follow emotional faces) and incongruent trials. If the RT in the congruent trials is shorter than that in the incongruent trials, it indicates that participants have an attentional bias toward expressions. Then a 2 (Group: high-AQ, low-AQ) × 2 (Expression: positive, negative) repeated measures ANOVA was performed for RTs of expressions (positive-neutral, negative-neutral), and an independent sample *t*-test was performed for RTs of expressions (negative–positive).

#### 2.6.2. Eye-tracking measurements

To explore the roles of the high-and low-AQ groups in the attention to the expressions, we collected three aspects of eye-movement data: (1) Time to first fixation: the time from when the expression was presented to the first fixation on the expression; (2) Fixation duration: the time from the first fixation on the expression to the gaze leaving the expression; (3) Fixation count: the number of fixations from the first fixation on the expression to the gaze leaving the expression.

According to previous studies (Gamble and Rapee, 2010; Kou et al., 2015), eye-movement data analysis mainly includes the following three indicators: (1) First fixation bias scores are the time to the first fixation on expressions (negative/positive) minus time to the first fixation on neutral faces. For example, a bias score for negative faces is the time to the first fixation on negative faces minus the time to the first fixation on negative–positive face pairs. A bias score below 0 indicates an accelerated detection bias toward emotional faces, a score equal to 0 indicates no bias, and a bias score above 0 indicates a deceleration detection bias toward emotional faces. (2) Fixation duration bias scores are the time to fixation on emotional faces as a proportion of the total fixation time for that face pair type. For example, a bias score for negative faces is the time to fixation on negative faces divided by the time to fixation in which eye movements are made toward negative–positive face pairs. A bias score above 0.5 indicates vigilance for emotional faces, a score equal to 0.5 indicates no attention bias, and a score below 0.5 indicates avoidance. (3) Fixation count bias scores are the number of fixations directed toward emotional faces as a proportion of total fixations on that face pair type. For example, a bias score for negative faces is the number of fixations directed toward negative faces divided by the number of valid fixations on negative–positive face pairs. A bias score above 0.5 indicates vigilance for the emotional face, a score equal to 0.5 indicates no attention bias, and a score below 0.5 indicates avoidance.

#### 2.6.3. Data analysis and statistics

First fixation scores were entered into a 2 (Group: high-AQ, low-AQ) × 2 (Expression: positive, negative) repeated measures ANOVA when the presented faces were positive-neutral pairs and negative-neutral pairs. Independent sample *t*-tests were conducted for the first fixation scores in the negative–positive pair trials. Referring to previous studies (Gamble and Rapee, 2010), the 8 s stimulus exposure was divided into eight time periods at 1 s intervals (0–1 s, 1–2 s, 2–3 s, 3–4 s, 4–5 s, 5–6 s, 6–7 s, and 7–8 s), and the eye-movement data of the two groups of subjects in different time periods were analyzed. Scores were entered into a 2 (Group: high-AQ, low-AQ) × 2 (Expression: positive, negative) × 8 (Time: 0–1 s, 1–2 s, 2–3 s, 3–4 s, 4–5 s, 5–6, 6–7 s, 7–8 s) repeated measures ANOVA with fixation duration scores and fixation count scores as dependent variables in the positive-neutral and negative-neutral pair trials. Scores were entered into a 2 (Group: high-AQ, low-AQ) × 8 (Time: 0–1 s, 1–2 s, 2–3 s, 3–4 s, 4–5 s, 5–6 s, 6–7 s, 7–8 s) repeated measures ANOVA with fixation duration scores and fixation count scores as dependent variables in the positive–negative pair trials.

## 3. Results

### 3.1. Behavioural data

The data from the positive-neutral and negative-neutral pair trials were entered into separate 2 (Group: high-AQ, low-AQ) × 2 (Expression: positive, negative) repeated measures ANOVAs with RT as the dependent variable. The main effects of the group [*F*(1, 48) = 0.07, *p* = 0.796, η^2^_p_ < 0.01] and expression [*F*(1, 48) = 1.03, *p* = 0.315,η^2^_p_ = 0.02] were not significant, and the interaction effect between the group and expression was not significant [*F*(1, 48) = 0.05, *p* = 0.824, η^2^_p_ = 0.001]. In the positive–negative pair trials, an independent sample *t*-test showed that the RTs of participants showed no significant differences (*t* = −1.46, *p* = 0.150).

### 3.2. Bias for first fixation

**Figure 2:**
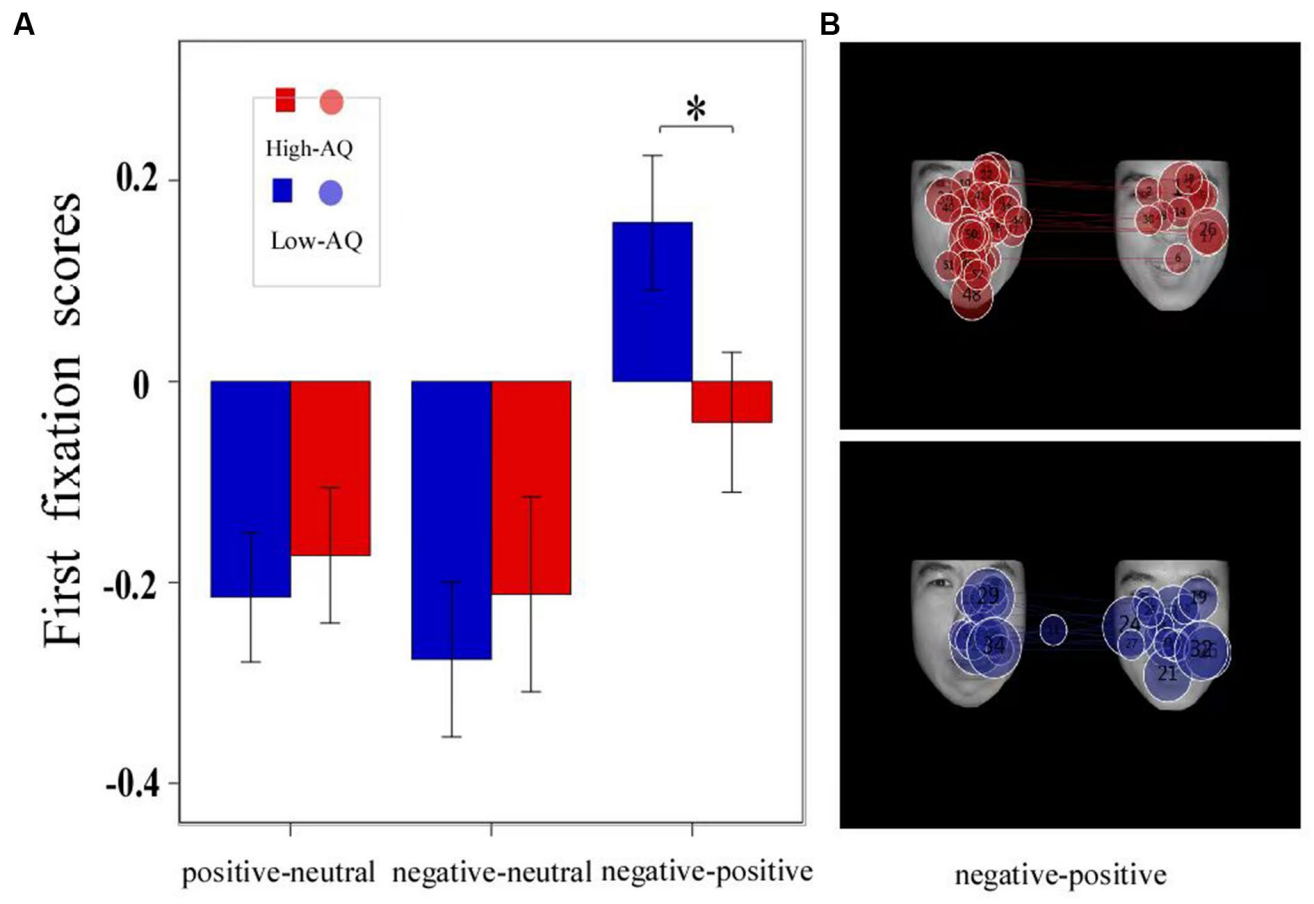
The bar charts and fixation trajectory graphs of first fixation scores made by groups in different facial expression pairs. Data in the bar charts are expressed as Mean ± SE. *: *p* < 0.05. **(A)** The first fixation scores of three Expressions. **(B)** The fixation trajectory graphs show the fixation trajectories of two groups of participants during 0–1 s in the negative-positive pairs trials, and the numbers in the circle indicate the order of the fixation count, with negative facial expression on the left and positive facial expression on the right.

The descriptive statistical results of first fixation scores are shown in Table 2, and the results of the variance test are shown in Table 3. The bar graph and fixation trajectory map of first fixation scores in the different face pair trials are shown in Figure 2.

**Table 2:** Statistical analysis results of first fixation scores for high-AQ group and low-AQ group (*M ± SD*).

Group	Positive-neutral	Negative-neutral	Negative–positive
High-AQ	−0.17 ± 0.33	−0.21 ± 0.48	−0.04 ± 0.34
Low-AQ	−0.21 ± 0.32	−0.28 ± 0.39	0.16 ± 0.33

**Table 3:** ANOVA results of first fixation scores in the positive-neutral and negative-neutral pair trials.

	*F*	*p*	η^2^_p_
Expression	0.49	0.488	0.01
Group	0.42	0.522	0.01
Expression × Group	0.03	0.873	< 0.01

First fixation bias scores were entered into a 2 (Group: high-AQ, low-AQ) × 2 (Expression: positive, negative) repeated measures ANOVA. In the positive-neutral and negative-neutral pair trials, there were no significant main effects of group [*F*(1, 48) = 0.42, *p* = 0.522, η^2^_p_ = 0.009] and expression [*F*(1, 48) = 0.49, *p* = 0.488, η^2^_p_ = 0.01]. The Group × Expression interaction was not significant [*F*(1, 48) = 0.03, *p* = 0.873, η^2^_p_ = 0.001].

In the positive–negative pair trials, an independent sample *t*-test showed that there was a significant difference in first fixation scores between the high-and low-AQ groups (*t* = 2.058, *p* = 0.045), and the first fixation scores of the high-AQ group (−0.04 ± 0.34) were significantly lower than those of the low-AQ group (0.16 ± 0.33).

### 3.3. Bias for fixation duration

**Figure 3:**
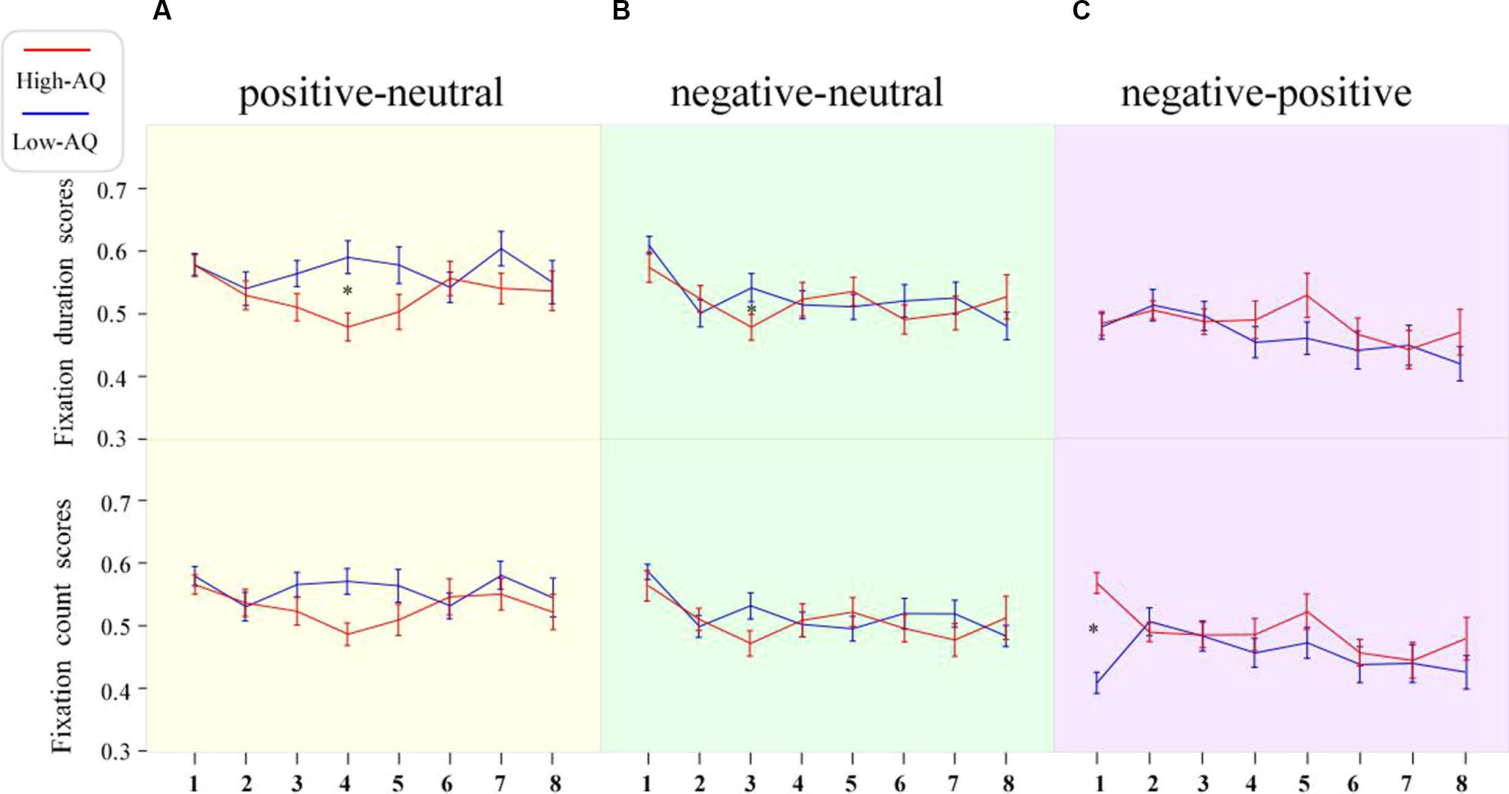
Line charts of participants’ fixation duration scores and fixation count scores in the different expression pair trials. The numbers 1–8 in the figure represent the scores of stimuli during 0–1 s, 1–2 s, 2–3 s, 3–4 s, 4–5 s, 5–6 s, 6–7 s, and 7–8 s, respectively. Data in the line charts are expressed as Mean ± SE.*: *p* < 0.05. **(A)** The scores of in the positive-neutral expression pair trials. **(B)** The scores of in the negative-neutral expression pair trials. **(C)** The scores of in the negative-positive expression pair trials.

**Figure 4:**
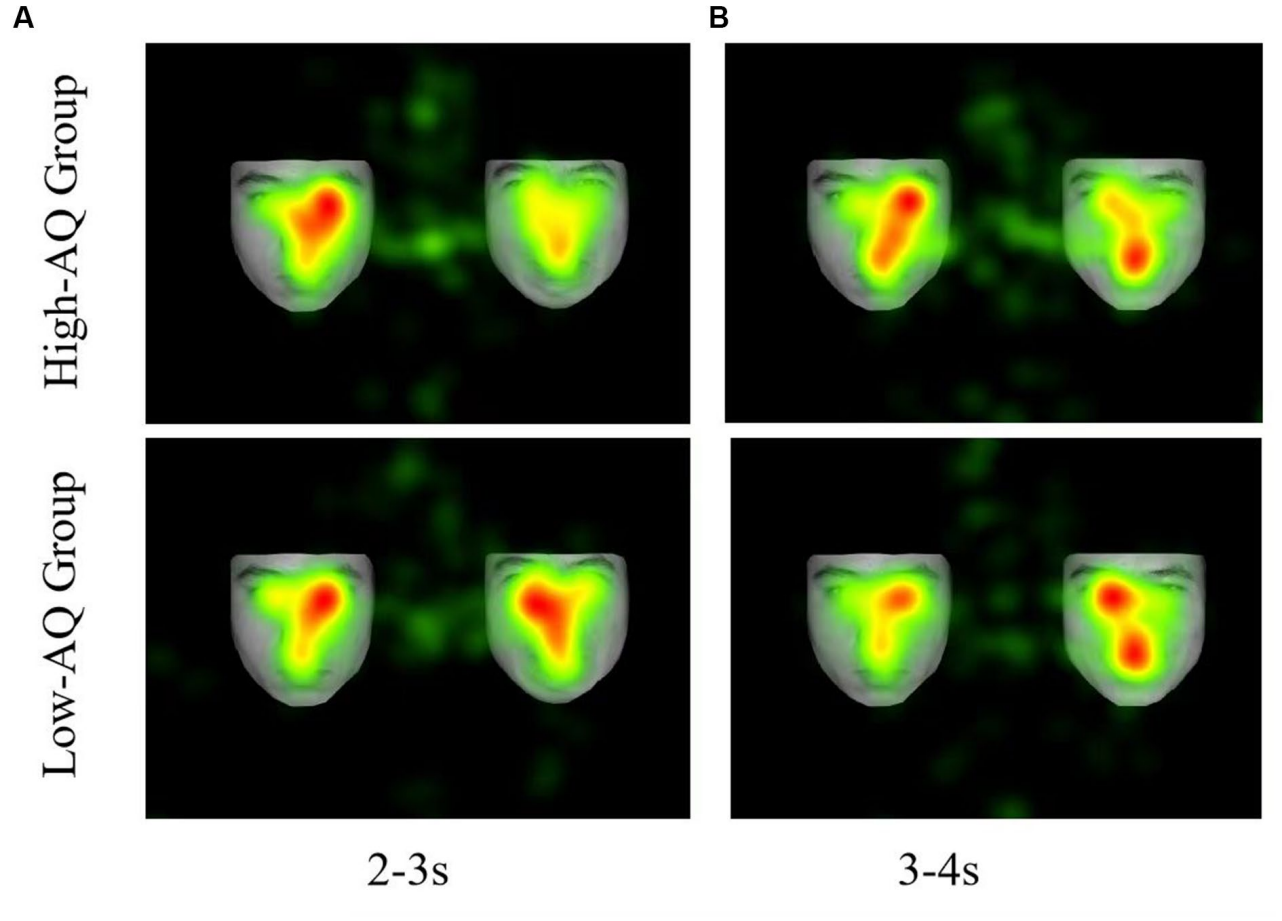
Fixation duration hotspot graph of participants in the different conditions. **(A)** The left side shows a neutral facial expression, and the right side shows a negative facial expression. **(B)** The left side shows a neutral facial expression, and the right side shows a positive facial expression.

Fixation duration bias scores were entered into a 2 (Group: high-AQ, low-AQ) × 2 (Expression: positive, negative) × 8 (Time: 0–1 s, 1–2 s, 2–3 s, 3–4 s, 4–5 s, 5–6 s, 6–7 s, 7–8 s) repeated measures ANOVA in the positive-neutral and negative-neutral pair trials (Table 4). There was a significant main effect of time as the total fixation duration scores of the subjects at 0–1 s were significantly higher than those in the other time periods [*F*(1, 48) = 4.04, *p* < 0.001, η^2^_p_ = 0.08]. The Group × Expression × Time interaction was significant [*F*(7, 42) = 2.28, *p* = 0.028, η^2^_p_ = 0.05]. Simple effect analysis showed that in the positive expressions trials [*F*(1, 48) = 10.50, *p* = 0.002, η^2^_p_ = 0.18], the total fixation duration scores of the high-AQ group (0.47 ± 0.11) were significantly lower than those of the low-AQ group (0.59 ± 0.13) during 3–4 s; in the negative expressions trials [*F*(1, 48) = 4.27, *p* = 0.044, η^2^_p_ = 0.08], the fixation duration scores of the high-AQ group (0.48 ± 0.10) were significantly lower than those of the low-AQ group (0.54 ± 0.11) during 2–3 s. In the negative expressions trials, there was a significant main effect of time in the high-AQ group [*F*(7, 42) = 3.02, *p* = 0.012, η^2^_p_ = 0.34], and the fixation duration scores of the high-AQ group during 0–1 s were significantly higher than those during 2–3 s, 5–6 s, and 6–7 s. However, in the positive expressions trials, the main effect of time was not significant [*F*(7, 42) = 2.01, *p* = 0.077, η^2^_p_ = 0.25]. The other main effects and interactions were not significant (*p* > 0.05). The fixation duration scores and fixation trajectory are shown in Figures 3, 4.

**Table 4:** Variance analysis results of fixation duration scores and fixation count scores in the positive-neutral and negative-neutral pair trials.

	Fixation duration scores			Fixation count scores			*F*	*p*	η^2^_p_	*F*	*p*	η^2^_p_
Expression	3.51	0.067	0.07	**4.61**	**0.037**	**0.09**
Time	**4.04**	**<0.001**	**0.08**	**3.96**	**< 0.001**	**0.08**
Group	2.05	0.158	0.04	1.69	0.200	0.03
Expression × Group	1.10	0.299	0.02	0.42	0.522	0.01
Time × Group	1.69	0.110	0.03	1.25	0.272	0.03
Expression × Time	1.23	0.284	0.03	1.19	0.309	0.02
Expression × Time × Group	**2.28**	**0.028**	**0.05**	1.86	0.075	0.04

df: (1, 48), The significant comparisons (*p* < 0.05) are shown in boldface.

First duration bias scores were entered into a 2 (Group: high-AQ, low-AQ) × 8 (Time: 0–1 s, 1–2 s, 2–3 s, 3–4 s, 4–5 s, 5–6 s, 6–7 s, 7–8 s) repeated measures ANOVA in the positive–negative pair trials (Table 5). The results showed that the main effects of time [*F*(1, 48) = 2.07, *p* = 0.065, η^2^_p_ = 0.04] and group [*F*(1, 48) = 0.085, *p* = 0.360, η^2^_p_ = 0.02] were not significant. Similarly, the interaction between time and group was not significant [*F*(1, 48) = 0.078, *p* = 0.575, η^2^_p_ = 0.02]. The fixation duration scores are shown in Figure 3.

**Table 5:** Variance analysis results of fixation duration scores and fixation count scores in the positive–negative pair trials.

	Fixation duration scores			Fixation count scores			*F*	*p*	η^2^_p_	*F*	*p*	η^2^_p_
Time	2.07	0.065	0.04	**2.30**	**0.042**	**0.05**
Group	0.85	0.360	0.02	1.69	0.200	0.03
Time × Group	0.78	0.575	0.02	**3.32**	**0.005**	**0.07**

df: (1, 48), The significant comparisons (*p* < 0.05) are shown in boldface.

### 3.4. Bias for fixation count

Fixation count bias scores were entered into a 2 (Group: high-AQ, low-AQ) × 2 (Expression: positive, negative) × 8 (Time: 0–1 s, 1–2 s, 2–3 s, 3–4 s, 4–5 s, 5–6 s, 6–7 s, 7–8 s) repeated measures ANOVA in the positive-neutral and negative-neutral trials (Table 4). The results showed that the main effect of expression was significant [*F*(1, 48) = 4.61, *p* = 0.037, η^2^_p_ = 0.09] and the total fixation count scores of participants for positive expressions (0.54 ± 0.08) were significantly greater than those for negative expressions. The main effect of time was significant [*F*(1, 48) = 3.96, *p* < 0.001, η^2^_p_ = 0.08], and the participants’ fixation count scores during 0–1 s were significantly higher than those in the other time periods. The other interactions were not significant (*p* > 0.05). The fixation count scores are shown in Figure 3.

Fixation count bias scores were entered into a 2 (Group: high-AQ, low-AQ) × 8 (Time: 0–1 s, 1–2 s, 2–3 s, 3–4 s, 4–5 s, 5–6 s, 6–7 s, 7–8 s) repeated measures ANOVA in the positive–negative pair trials (Table 5). The main effect of time was significant [*F*(1, 48) = 2.30, *p* = 0.042, η^2^_p_ = 0.05]. The interaction between time and group was significant [*F*(1, 48) = 3.32, *p* = 0.005, η^2^_p_ = 0.07]. The simple effect analysis found that the fixation count scores of the high-AQ group (0.57 ± 0.08) were significantly higher than those of the low-AQ group (0.41 ± 0.08, *p* < 0.001) during 0–1 s. The other main effects and interactions were not significant (*p* > 0.05). The fixation count scores are shown in Figure 3.

## 4. Discussion

The study used eye-tracking technology to investigate the time course of attentional bias toward emotional faces in individuals with ATs. The results showed that the high-AQ group exhibited attentional vigilance toward emotional faces, detecting negative emotional faces faster than the low-AQ group in the early stage of emotional face processing. The fixation scores of the high-AQ group were significantly lower than those of the low-AQ group during the presentation of negative-neutral faces at 2–3 s and positive-neutral faces at 3–4 s, with fixation scores of the high-AQ group all less than 0.5. Therefore, as the presentation time of emotional faces increased, the high-AQ group showed attentional avoidance toward both positive and negative emotional faces compared with the low-AQ group, with attentional avoidance toward negative emotions occurring earlier than that of positive emotions. The above results support the Intense World Theory.

The first fixation scores show the detection speed of individuals for emotional faces (Kou et al., 2015). The results indicate that the high-and the low-AQ groups showed accelerated detection bias for positive and negative expressions compared to neutral faces in both the positive-neutral and negative-neutral trials, and there was no significant difference in detection speed between the two groups of participants. This indicates that when compared with neutral faces, the two groups can prioritize the detection of emotional faces and demonstrate attentional vigilance to emotional faces. The high-AQ group showed an accelerated detection bias for negative emotional faces in the negative–positive trials, while the low-AQ group did not have an attention bias. This suggests that individuals with ATs are more attentionally vigilant to negative emotional faces compared with neutral and positive emotions; this is consistent with previous research findings (Zhao et al., 2016). The fixation count scores of the high-AQ group at 0–1 s were significantly higher than those of the low-AQ group in the negative–positive trials, and no significant differences were observed in the remaining time periods. Moreover, there were no significant differences in fixation duration at 0–1 s between the two groups, indicating that the high-AQ group had a higher frequency of gazing at negative emotional faces in the early stage than the low-AQ group. Additionally, the first fixation scores of the high-AQ group were significantly higher than those of the low-AQ group. The results revealed that the high-AQ group had a significantly faster detection speed for negative emotional faces than the low-AQ group when positive and negative emotional faces were presented simultaneously. This result is similar to the findings of studies conducted with individuals with ASD. Some studies have found that individuals with ASD have a detection advantage for negative emotional faces, detecting negative emotional faces faster than the general population (Isomura et al., 2014; May et al., 2015). Therefore, participants exhibit attention vigilance toward facial expressions in the early stages of emotional facial processing, but the high-AQ group had a significantly faster detection speed for negative emotions than the low-AQ group. Attention vigilance is based on the amygdala-early visual cortex, which is a bottom-up direct response system (Davis and Whalen, 2001). The Intense World Theory proposes that there are excessive connections between brain neurons and excessive activation in certain brain regions (e.g., the prefrontal cortex and sensory cortex) and the amygdala in individuals with ASD (Markram and Markram, 2010). Therefore, the excessive activation of these regions allows attention resources to be quickly allocated to negative emotional stimuli, which makes individuals with ATs more alert to negative emotions and faster in detecting them than the low-AQ group.

In the positive-neutral and negative-neutral trials, there was a significant interaction between expression, time, and group on the fixation duration scores. Compared with the low-AQ group, the high-AQ group only showed brief attentional avoidance toward positive emotion at 3–4 s in the positive-neutral trials, and there was no attentional avoidance in the other time periods. Moreover, some studies have found that individuals with ASD may show attentional avoidance toward positive emotions (Zhao et al., 2016). At the same time, the ASD group displayed attentional avoidance toward negative expressions earlier than toward positive expressions, suggesting that negative emotional faces may be more likely to induce sensory overload. The fixation scores for negative-neutral faces of the high-AQ group during 2–3 s were less than 0.5, which is significantly lower than those of the low-AQ group, indicating that the high-AQ group exhibited significant attentional avoidance toward negative expressions compared with the low-AQ group during this time interval. Our study results are similar to those of previous studies on individuals with ASD: both show attentional bias toward negative expressions and may avoid such faces under prolonged stimulus presentation (García-Blanco et al., 2017). Recent research on the attention patterns of individuals with ASD toward emotional faces has found that the directional attention of individuals with ASD toward negative emotional information may cause them to have overly intense emotional reactions and, therefore, avoid such strong stimuli (Ghosn et al., 2019). The Intense World Theory suggests that overly active brain regions greatly amplify ordinary local sensory experiences, which places individuals with ASD in a state of sensory information overload and causes them to experience excessive fear and anxiety toward social stimuli such as faces, leading to the emergence of attentional avoidance (Sahuquillo-Leal et al., 2023). Therefore, the attentional avoidance of individuals with ATs may be related to their sensory overload.

Interestingly, we found that the high-AQ group showed a dynamic change in attentional patterns to negative expressions in the negative-neutral trials: they exhibited attention maintenance during the face presentation at 0–2 s, 3–5 s, and 7–8 s and attention avoidance during the face presentation at 2–3 s and 5–7 s. By contrast, the low-AQ group showed attention maintenance toward negative expressions until 7 s and then exhibited attention avoidance. Therefore, the high-AQ group showed earlier attentional avoidance to negative expressions compared with the low-AQ group in the middle to late stages of attentional processing. A study found that individuals with ASD had significantly shorter fixation durations on negative expressions in the last 3–4 s of emotional face presentation (White et al., 2015), indicating a decline in attention toward negative emotional faces in the later stages. At the same time, based on the attention process of individuals with ATs to negative emotional faces, we speculate that the duration of attention maintenance to negative emotional faces in the high-AQ group may be approximately 2 s, and attention avoidance occurs after 2 s; the duration of attention avoidance will increase with the presentation time of the faces. There is a dynamic change in the attention of the high-AQ group toward negative emotional faces, which may involve both bottom-up and top-down attention processes. On the one hand, compared with neutral faces, negative faces may more easily capture the attention of individuals with ATs; on the other hand, these individuals may experience sensory overload when processing negative emotional information. To avoid discomfort caused by sensory overload (e.g., excessive anxiety or fear), individuals with ATs engage in top-down self-regulation, shifting their attention to neutral faces and avoiding negative emotional faces. As seen in studies on individuals with ASD, to avoid discomfort and threat posed by direct eye contact, they may shift their attention to the mouth area (Neumann et al., 2006). However, the self-regulation mechanism in individuals with ATs may be influenced by hyperactivation of certain areas of the cortex, leading to a brief avoidance of negative emotional faces, followed by a return to the direction of attention toward these faces. Therefore, the attention pattern of individuals with ATs toward negative emotional faces exhibits a dynamic change.

In summary, in the early stages of emotional face processing, the high-and low-AQ groups exhibited attentional vigilance to both positive and negative emotional faces compared with neutral faces. When comparing negative emotional faces to positive emotional faces, the high-AQ group exhibited attentional vigilance to negative expressions and detected them faster than the low-AQ group. As the time of facial stimuli increased, individuals with ATs showed greater attentional avoidance toward both positive and negative emotional faces than the low-AQ group. However, the attention avoidance of negative expressions was earlier and longer than that of positive expressions. These results extend the attentional similarities between individuals with ASD and ATs and provide important evidence for the facial processing characteristics of individuals with ATs. In addition, the executive attention network is related to the anterior cingulate gyrus, ventral striatum, prefrontal cortex, and basal ganglia (Posner et al., 2006), and the attention pattern of ATs may be related to the dysfunction of this network. Increasing evidence shows that ATs have a related neural structural basis; they are related to the connection of white matter fibers in the brain: positively related to the connection between the superior temporal sulcus and the amygdala (Lui et al., 2018) and negatively related to the functional connection between the cingulate cortex and the anterior insular lobe (Martino et al., 2009). Based on this, excessive connections between neurons or overreactions in brain regions may cause individuals with ATs to exhibit a vigilance-avoidance attention pattern toward emotional faces. Therefore, this study also provides a basis for further exploration of the neural basis of attention characteristics in groups with ATs. In the future, we can combine neuropsychology to study executive dysfunction and cortical abnormalities, which will not only deepen our understanding of the neuropsychological characteristics of ATs but also promote gene research on ATs through the association of specific cognitive functions with ATs.

## Data availability statement

The original contributions presented in the study are included in the article/Supplementary material, further inquiries can be directed to the corresponding author.

## Ethics statement

The studies involving human participants were reviewed and approved by research ethics committee of Chongqing Normal University. The patients/participants provided their written informed consent to participate in this study.

## Author contributions

CM and JW designed the experiments and collected data. CM conducted the eye-tracking data analysis. TL and CM completed the statistical analysis and wrote the manuscript. TL and JW revised the manuscript. All authors contributed to the article and approved the submitted version.

## Funding

This study was supported by grants from the Nanchong Social Science Research “14th Five-Year Plan” (NC23B123).

## Conflict of interest

The authors declare that the research was conducted in the absence of any commercial or financial relationships that could be construed as a potential conflict of interest.

## Publisher’s note

All claims expressed in this article are solely those of the authors and do not necessarily represent those of their affiliated organizations, or those of the publisher, the editors and the reviewers. Any product that may be evaluated in this article, or claim that may be made by its manufacturer, is not guaranteed or endorsed by the publisher.
