# Supplementary material for: Temporal course of attention bias toward emotional faces in individuals with autistic traits: an eye-movement study
Source: Front Neurosci. 2023 Jul 28;17:1218595. doi: 10.3389/fnins.2023.1218595 (PMC10416432; doi:10.3389/fnins.2023.1218595)
Supplement: Supplementary file 1 [file Table_1.docx]

Supplementary Material

The temporal-course of attention bias to emotional faces in autistic traits: An eye-movement study

Chunyan Meng*, Taolin Li, Jing Wang

*** Correspondence:** Chunyan Meng: [mcy77852022@163.com](mailto:mcy77852022@163.com)

# Supplementary Tables

Table1 Performance of Classification and Summary in 19 studies

|  | **References** | **Participants** | **Paradigm** | **Technology** | **Stimulus** | **Main findings** | **Exist problems** |
| --- | --- | --- | --- | --- | --- | --- | --- |
| ASD | Papagiannopoulou et al., 2014 | children  with ASD | — | meta-analysis | face（eyes mouth ） | Children with ASD have significantly reduced gaze fixation to the eye region of faces. The results of the meta-analyses indicate that ASD patients have significant impairments in gaze fixation to the eyes. | This study mainly focuses on the gaze of children with ASD on the facial area, without studying adults with ASD. At the same time, no research has been conducted on faces with different emotions. |
|  | Hao et al., 2018 | ASD | — | meta-analysis | face（eyes，mouth ） | ASD individuals view the eyes as a threatening stimulus, focusing less on the eyes than the general population, and there is no difference in focusing on the mouth, thus supporting the hypothesis of eye threat anxiety. | The article focuses more on the gaze of ASD individuals on facial areas (eyes, mouth, etc.), without incorporating different emotional faces. |
|  | García-Blanco et al., 2017 | children with ASD and TD | dot-probe | behavioral studies | facial expressions  (happy, angry, and sad , neutral ) | When compared to the TD group, the ASD group showed an attentional bias away from angry faces at long presentation rates, and did not exhibit a significant attention bias towards happy faces | This study explored the attention bias of ASD children towards different emotional faces, mainly analyzing behavioral data of attention bias in ASD children, but did not further capture the specific attention bias trajectory and time course of ASD children. |
|  | Hollocks et al., 2016 | boys with  ASD | dot-probe | behavioral studies | face pairs (angry-neutral, happy-neutral， neutral–neutral ) | The study found that there were significant group differences in attentional bias towards threatening, but not happy faces. The ASDanx group had a significantly greater threat bias towards angry faces compared  to both the control group. | The study did not include a non-ASD anxiety disorder group. This would have allowed for a direct comparison of task performance and provide more substantial evidence of either similar or different cognitive and physiological mechanisms in anxiety with and without ASD. |
|  | Markram et al., 2010 | ASD | — | literature review | — | The Intense World Theory of autism proposes excessive functioning of neural microcircuits, with the main symptoms being hyper-reactivity and hyper-plasticity and together, hyper-functionality | Lack of further experimental verification |
|  | Sahuquillo-Leal, et al., 2023 | individuals with ASC and TD | free-  viewing | eye movements | emotional scenes (happy, neutral, threatening, sad) | Perceived threat induces an early overwhelming response in autism, giving rise to an avoidance behavior | The findings may not be generalizable to low-functioning ASC individuals. |
|  | Monk et al., 2010 | ASD | attention cuing | functional MRI | emotional faces (happy, sad, angry, neutral) | When attention bias to emotional faces was equivalent between ASD and control groups, ASD was associated with greater amygdala activation. | Lack of congruence in stimuli when emotional faces were presented. |
|  | Chen et al., 2011 | Children with ASD and TD | dot-probe | eye movements | landscape images and emotional faces (neutral, fear, happy) | ASD children have a longer detection time for faces with different emotions than normal children, a shorter processing time for faces, and longer fixation time for fearful faces than for neutral or happy faces | This article only focuses on characteristic research and lacks research on neural mechanisms |
|  | Isomura et al., 2014 | Children with ASD and TD | Search-  Recognition | — | facial expressions(angry, happy, neutral) | Children with ASD may extract emotional information from local features in angry faces and showed the proper emotional response of detecting angry faces over happy faces. | The neural basis of different mechanisms in children with TD and ASD |
|  | Ghosn et al., 2019 | Children with ASD and TD | dot-probe | behavioural studies | static social scenes(happy, neutral,sad,threatening) and emotional face (angry, happy, sad, neutral) | ASD children showed an attentional bias toward threatening scenes but away from threatening faces. | Research on the neural mechanisms by which threatening social scenes trigger attention alertness and threatening facial expressions lead to attention avoidance. |
|  | Fan et al., 2020 | HFA | — | literature review | emotional faces | In the control processing stage, where tasks are unrelated to emotions, they demonstrate threatening emotional face attention bias. | Further explore the relevant processing characteristics and neuro-biological mechanisms and make efforts to develop scientific and effective intervention strategies. |
|  | White et al., 2015 | adolescents with ASD and without ASD | — | eye movements | facial expression | Among cognitively unimpaired adolescents with ASD, self-reported fear of negative evaluation predicted greater gaze duration to social threat cues | Expand research on potential social influencing factors of individuals with autism (such as alexithymia and depression) |
| TA | Folz et al., 2023 | healthy participants | dot-probe | behavioural studies | emotional expressions | Less pronounced bias towards happy facial expressions with higher autistic trait levels. Moreover, a closer examination of the attentional bias towards angry facial expressions suggested that alterations in this bias might depend on a complex interplay between both trait dimensions. | Novel approaches in the assessment of attentional biases might yield the potential to describe disorder-specific biases in attention to emotions more validly |
|  | Greene et al., 2020 | adults | dot-probe | eye movements | emotional faces | AT in the general population do not affect visual processing of emotional faces. | More complex social situations may be necessary to reveal an influence of AT on emotional face processing |
|  | Miu et al., 2012 | low AT high AT | RMET | — | emotional faces (neutral ,fearful) | High AT participants displayed enhanced observational FC, no attentional bias to fearful faces, and increased latency (but normal accuracy) to recognizing the mental state of another. | Increasingly focus on general population samples selected for extreme AT, in order to increase the relevance of results for the broad autism phenotype perspective |
|  | Freeth et al., 2013 | healthy participants | — | eye-tracking | face-to-face, interaction ;  pre-recorded video interaction | The study found increased autistic traits were associated with less looking at the experimenter for video interactions . | These two studies found that individuals with higher scores of autism traits have less eye contact in face-to-face and video interactions, but did not further investigate the eye contact responses of individuals with autism traits to different emotional faces. |
|  | Vabalas et al., 2016 | low BAPQ high BAPQ | **—** | eye-tracking | face-to-Face interaction | The study found individuals who  were high in autistic traits exhibited reduced visual exploration during the face-to-face interaction overall, as  demonstrated by shorter and less frequent saccades. |  |
|  | Chen et al., 2010 | low AQ  high AQ | **—** | eye-tracking | naturalistic videos | The study found Individuals with lower scores on four subscales of the AQscale showed a greater tendency to look at directed relative to averted eyes; individuals with higher scores on the AQ did not. | The article only studied the gaze of the participants(Low AQ and High AQ) towards the eyes, without paying attention to the reactions of the subjects under different emotional and facial stimuli. |
|  | Lassalle et al., 2015 | healthy participants | gaze-cueing | ERPs | dynamic face(fearful ,happy faces） | The study found that the GOE was larger for fearful than happy faces but only in participants with high AQ scores. Autistic traits influence attention orienting to gaze and its modulation by social emotions such as happiness. | The study did not observed influence of autistic traits on the brain processes devoted to attention. |

MRI: Magnetic Resonance Imaging；ASD: Autism Spectrum Disorder; ASC: Autism Spectrum Condition ；TD: Typically Developed; HFA: High-Functioning Autism;  RMET : Reading the Mind in the Eyes Test; AQ: Autism Quotient; FC: fear conditioning; BAPQ: Broad Autism Phenotype Questionnaire; GOE: Gaze Orienting Effect.

**
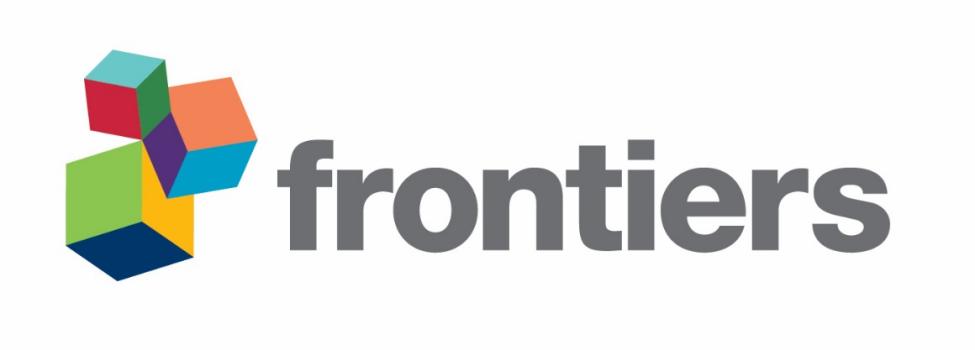
**
